# MNK1 and MNK2 enforce expression of E2F1, FOXM1, and WEE1 to drive soft tissue sarcoma

**DOI:** 10.1038/s41388-021-01661-4

**Published:** 2021-02-09

**Authors:** Xin-Yu Ke, Ye Chen, Valarie Yu-Yan Tham, Ruby Yu-Tong Lin, Pushkar Dakle, Kassoum Nacro, Mark Edward Puhaindran, Peter Houghton, Angela Pang, Victor Kwanmin Lee, Ling-Wen Ding, Sigal Gery, Jeffrey Hill, Leilei Chen, Liang Xu, H. Phillip Koeffler

**Affiliations:** 1 Cancer Science Institute of Singapore, National University of Singapore, Singapore, Singapore; 2 Department of Anatomy, Yong Loo Lin School of Medicine, National University of Singapore, Singapore, Singapore; 3 Experimental Drug Development Centre, Agency for Science, Technology and Research, Singapore, Singapore; 4 National University Cancer Institute, National University Hospital, Singapore, Singapore; 5 Division of Musculoskeletal Oncology, University Orthopaedics, Hand and Reconstructive Microsurgery Cluster, National University Hospital, Singapore, Singapore; 6 Department of Hand and Reconstructive Microsurgery, National University Hospital, Singapore, Singapore; 7 Greehey Children’s Cancer Research Institute, UT Health San Antonio, San Antonio, TX USA; 8 Department of Pathology, National University Hospital, Singapore, Singapore; 9 Department of Pathology, Yong Loo Lin School of Medicine, National University of Singapore, Singapore, Singapore; 10 Division of Hematology/Oncology, Cedars-Sinai Medical Center, Los Angeles, CA USA; 11 Sussex Drug Discovery Centre, School of Life Sciences, University of Sussex, Brighton, UK

**Keywords:** Sarcoma, Target identification

## Abstract

Soft tissue sarcoma (STS) is a heterogeneous disease that arises from connective tissues. Clinical outcome of patients with advanced tumors especially de-differentiated liposarcoma and uterine leiomyosarcoma remains unsatisfactory, despite intensive treatment regimens including maximal surgical resection, radiation, and chemotherapy. MAP kinase-interacting serine/threonine-protein kinase 1 and 2 (MNK1/2) have been shown to contribute to oncogenic translation via phosphorylation of eukaryotic translation initiation factor 4E (eIF4E). However, little is known about the role of MNK1/2 and their downstream targets in STS. In this study, we show that depletion of either MNK1 or MNK2 suppresses cell viability, anchorage-independent growth, and tumorigenicity of STS cells. We also identify a compelling antiproliferative efficacy of a novel, selective MNK inhibitor ETC-168. Cellular responsiveness of STS cells to ETC-168 correlates positively with that of phosphorylated ribosomal protein S6 (RPS6). Mirroring MNK1/2 silencing, ETC-168 treatment strongly blocks eIF4E phosphorylation and represses expression of sarcoma-driving onco-proteins including E2F1, FOXM1, and WEE1. Moreover, combination of ETC-168 and MCL1 inhibitor S63845 exerts a synergistic antiproliferative activity against STS cells. In summary, our study reveals crucial roles of MNK1/2 and their downstream targets in STS tumorigenesis. Our data encourage further clinical translation of MNK inhibitors for STS treatment.

## Introduction

Soft tissue sarcoma (STS) is a heterogeneous neoplasm with more than 70 subtypes [1, 2]. Liposarcoma (LPS) and leiomyosarcoma (LMS) represent two most frequent subtypes of STS in adult [3, 4], while rhabdomyosarcoma (RMS) is the most common STS of children [5]. These three types of STS show histological presentation resembling partially the differentiation features of adipocytes, smooth muscle cells, and skeletal muscle cells, respectively. Current therapeutic modalities of STS mainly involve surgery, radiation, and chemotherapy; however, outcomes of patients with advanced STS remain unsatisfactory. More efforts are needed to explore alternative therapeutic options.

MAP kinase-interacting serine/threonine-protein kinase 1 and 2 (MNK1/2) are two downstream kinases of p38 MAPK and MAPK/ERK kinase-extracellular signal-regulated kinase (MEK-ERK) pathways, with their best-studied function to phosphorylate eukaryotic translation initiation factor 4E (eIF4E) at Ser^209^ site [6, 7]. To date, the oncogenic roles of MNK1/2 and phosphorylated eIF4E have been implicated in various cancers. For instance, depletion of MNK1/2 in KIT-mutant melanoma cells inhibited tumor progression through blocking translation of SNAI1 and CCNE1 [8]. Intriguingly, suppression of either MNK or eIF4E phosphorylation is well-tolerated without inhibitory effects on global translation. Mice with either single or double knockout of MNK1 and MNK2 were viable and born without abnormalities [9]. Consistent with these findings, small-molecule inhibitors targeting MNK1/2 (e.g., CGP57380, BAY1143269, eFT508, and SEL201) demonstrated promising anticancer efficacy and desirable safety [10]. Among these compounds, CGP57380 is widely used as a tool compound of MNK inhibitor [11, 12]. BAY1143269 inhibited MNK1 activity and its downstream factors including genes involved in cell cycle, survival, immune response, and epithelial–mesenchymal transition [13]. MNK1/2 inhibitor eFT508 is currently under clinical trials in hematological malignancies [14]; it also inhibited the translation of PD-L1 in a murine liver cancer model which was driven by MYC and KRAS [15]. Notably, apart from eIF4E-dependent oncogenic translation, MNK1/2 exert diverse roles in cancer-promoting pathways. MNK1/2 can phosphorylate a number of downstream substrates such as hnRNPA1 [16] and SPRY2 [17, 18]. Active MNK1/2 engaged TELO2 to sustain mTOR1 activity and contributed to rapamycin resistance in cancer cells [19]. In addition, MNK1/2 enhanced transcription of ANGPTL4 through peroxisome proliferator-activated receptor in melanoma cells [20]. In contrast to their well-established role in translation, impact of MNK1/2 on oncogenic transcription remains poorly explored.

Both RAS-MAPK and PI3K-AKT-mTOR signalling pathways are aberrantly elevated in STS, especially de-differentiated LPS and uterine LMS [21, 22]. Crosstalk between MNK1/2 and these two pathways implicates MNK1/2 as a common targetable downstream node in heterogeneous STS. The current study investigated functional relevance of MNK1/2 and their downstream targets in STS. By both genetic and pharmacological approaches, we demonstrated the essential role of MNK1 and MNK2 in soft-tissue sarcomagenesis, and identified E2F1, FOXM1, and WEE1 as critical downstream effectors of MNK1/2. Importantly, we also reported potent antiproliferative properties of a novel MNK inhibitor ETC-168 against STS cells. Co-inhibition of MNK1/2 and MCL1 yielded further a synergistic anti-STS activity. Together, our study reveals a targetable oncogenic dependency of STS cells on MNK1/2, providing rationale for further development of MNK1/2-targeted therapy.

## Results

### Elevated expression of MNK1 and MNK2 in STS cells

**Fig. 1:**
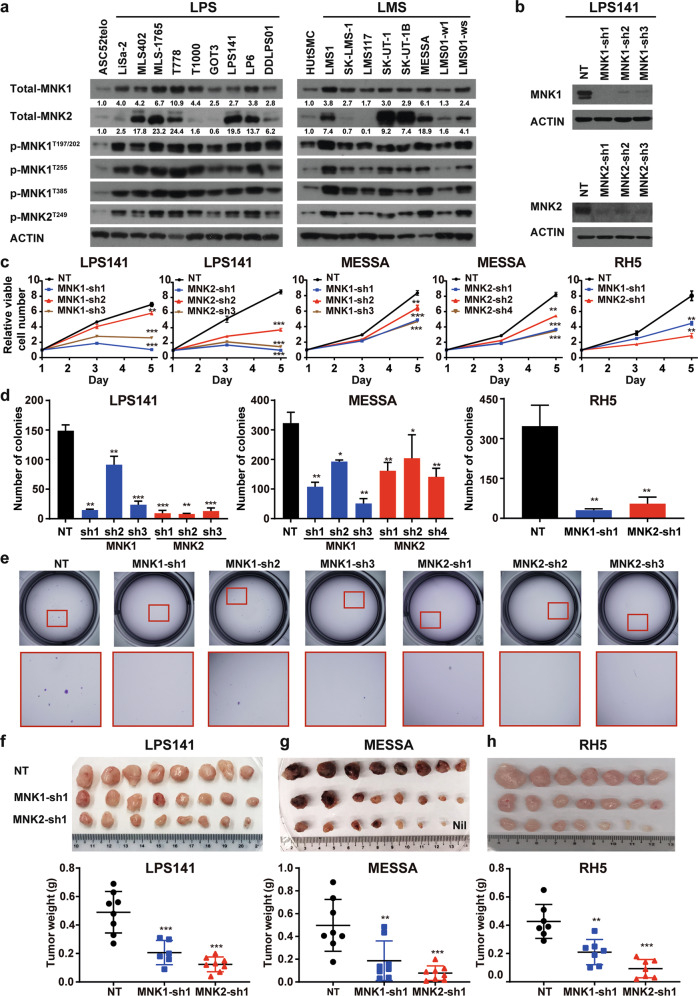
MNK1 and MNK2 are required for the growth of STS cells both in vitro and in vivo. **a** Western blot showing MNK1 and MNK2 protein expression in STS cells. **b** Verification of shRNA-mediated knockdown of MNK1 and MNK2 by western blot in LPS141 cells. Effects of MNK1 and MNK2 silencing on **c** cell viability and **d** anchorage-independent growth of LPS141, MESSA, and RH5 cells. **e** Defective colony formation of LPS141 cells in soft agar upon MNK1/2 knockdown. Effects of MNK1 and MNK2 ablation on in vivo tumor formation of **f** LPS141 (*n* = 8), **g** MESSA (*n* = 8), and **h** RH5 (*n* = 7) cells. Cells were subcutaneously injected into the upper flanks of NSG mice. After 3–4 weeks, xenograft tumors were harvested, weighed, and photographed. Nil, no tumor growth. Data of **c**, **d** are shown as mean ± SD from representative data out of three independent experiments. Statistical significance is determined by unpaired two-tailed Student *t* test. n.s. not significant; *p < 0.05; **p < 0.01; ***p < 0.001.

Expression and role of MNK proteins remain uncharacterized in STS cells, although overexpression of either MNK1 or MNK2 has been reported in several human malignancies [8, 23, 24]. To fill in the gap, we first examined protein expression of MNK1 and MNK2 in a panel of STS cell lines, including nine LPS cell lines (pleomorphic LPS: LiSa-2; myxoid LPS: MLS402, MLS-1765; well-differentiated LPS: T778, T1000, GOT3; de-differentiated LPS: LPS141, LP6, Shef-DDLPS01) and eight LMS cell lines (bone LMS: LMS1; vulvar LMS: SK-LMS-1; uterine LMS: LMS117, SK-UT-1, SK-UT-1B, MESSA; soft-tissue LMS: Shef-LMS01-w1, Shef-LMS01-ws). hTERT immortalized adipose-derived mesenchymal stem cells ASC52telo and human primary uterine smooth muscle cells HUtSMC were used as normal controls. MNK1 protein was moderately elevated in most STS cells in comparison with nonmalignant cells. MNK2 showed a more pronounced expression in about half of cancer cells than their respective nonmalignant counterparts (Fig. 1a). In addition, phospho-MNK1 (at Thr197/202, Thr255, and Thr385 sites) and phospho-MNK2 (at Thr249 site) were markedly increased across cancer cells, relative to ASC52telo and HUtSMC cells (Fig. 1a). These results reveal an enhanced expression of total and phospho-MNK proteins in STS cells.

### MNK1 and MNK2 confer a proliferative potential on STS tumorigenesis

To explore functional relevance of MNK1 and MNK2 in STS cells, shRNA-mediated gene silencing approach was employed to modulate their expression. Depletion of either MNK1 or MNK2 markedly reduced the cell viability of LPS141 (de-differentiated LPS), MESSA (uterine LMS), and RH5 (RMS) cells (Fig. 1b, c and Supplementary Fig. S1a, b). Consistently, defective expression of either MNK1 or MNK2 compromised colony-forming capability of these STS cells (Fig. 1d, e). Moreover, simultaneous targeting of both MNK1 and MNK2 yielded an additive growth-inhibitory effect on both cell viability and clonogenicity (Supplementary Fig. S1c, d). Next, we examined involvement of MNK1/2 in STS tumor growth in vivo. STS cells stably expressing shRNAs against either MNK1 or MNK2 developed significantly smaller xenografts in NOD scid gamma (NSG) mice (Fig. 1f–h). Together, our observations indicate that both MNK1 and MNK2 enforce growth of STS in vitro and in vivo.

### MNK kinase inhibitor ETC-168 exerts a potent antiproliferative effect

**Fig. 2:**
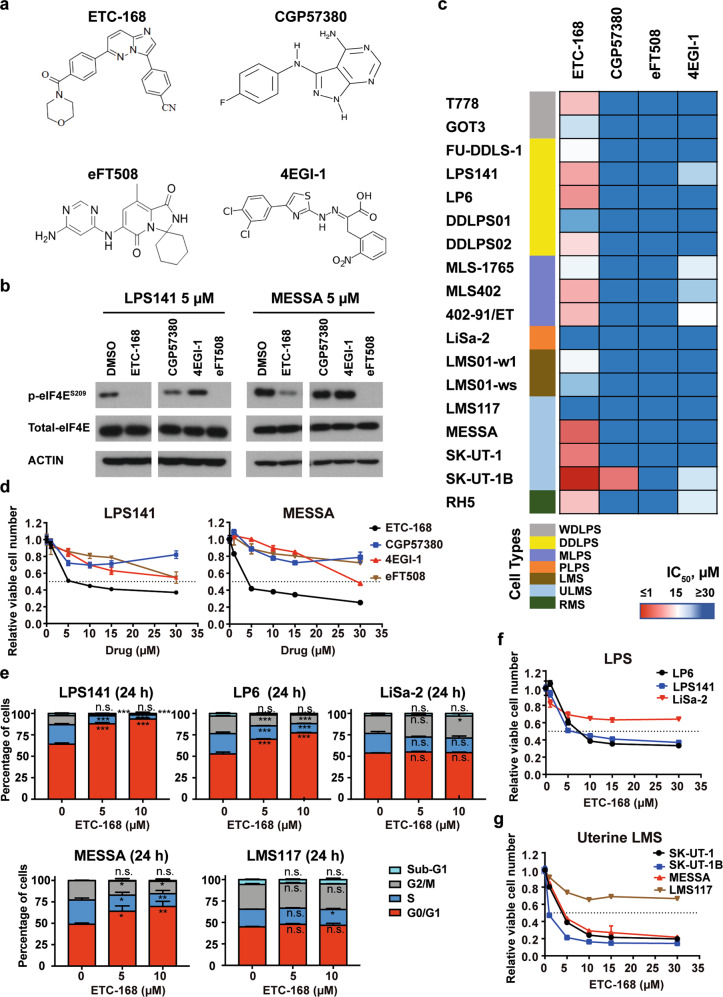
MNK inhibitor ETC-168 exerts a potent antiproliferative activity against STS cells. **a** Chemical structure of MNK inhibitors ETC-168, eFT508, and CGP57380, as well as eIF4E/eIF4G inhibitor 4EGI-1. **b** Effects of MNK/eIF4E inhibitors (5 μM, 24 h) on the protein expression of eIF4E in LPS141 and MESSA cells. **c** Heatmap showing antiproliferative activities of ETC-168, CGP57380, eFT508, and 4EGI-1 in 18 STS cell lines. WDLPS well-differentiated LPS, DDLPS de-differentiated LPS, MLPS myxoid LPS, PLPS pleomorphic LPS, LMS leiomyosarcoma, ULMS uterine LMS, RMS rhabdomyosarcoma. IC_50_ values were determined by MTT assay after 72-h treatment based on at least three independent repeats. **d** Dose-dependent curves showing the response of LPS141 and MESSA cells to ETC-168, CGP57380, 4EGI-1, and eFT508 treatment. **e** Cell cycle analysis of LPS cells (LPS141, LP6, and LiSa-2) and uterine LMS cells (MESSA and LMS117) in response to ETC-168 treatment (24 h). Dose-dependent curves showing the response of **f** LPS cells (LP6, LPS141 and LiSa-2) and **g** uterine LMS cells (SK-UT-1, SK-UT-1B, MESSA, and LMS117) to ETC-168. Data of **d**–**g** are shown as mean ± SD from representative data out of three independent experiments. Statistical significance of **e** is determined by one-way ANOVA. n.s. not significant; *p < 0.05; **p < 0.01; ***p < 0.001.

Functional importance of MNK1/2 prompted us to investigate whether MNK1/2 can be effective targets for STS treatment. To this end, we studied a novel MNK kinase inhibitor ETC-168 whose biochemical IC_50_ values against MNK1 and MNK2 were as low as 23 and 43 nM, respectively [25]. At the dose of 1 μM, ETC-168 achieved desirable inhibition of the kinase activity of MNK1 (58%) and MNK2 (97%), suggesting that ETC-168 acts as a MNK2-biased, dual-MNK inhibitor in cells. We screened the antiproliferative efficacy of ETC-168 against a large panel of 18 STS cell lines and compared with three additional agents targeting either MNK1/2 (i.e., CGP57380 and eFT508) or eIF4E activities (4EGI-1) [26] (Fig. 2a, b). In general, ETC-168 showed a greater potency than the rest of the agents (Fig. 2c). The concentrations of half maximal inhibition of cell proliferation of ETC-168 were less than 10 μM in most cell lines other than LiSa-2, LMS117, and the nonmalignant ASC52telo cells (Fig. 2c and Supplementary Fig. S2a). For instance, in LPS141 and MESSA, ETC-168 induced dose-dependent growth suppression and inhibited 50% of cell viability at a dose of 5 μM, while other inhibitors were less active (Fig. 2d). ETC-168 treatment elicited a consistent increase of cells in G0/G1 phase among LPS141, LP6, and MESSA cells in a dose-dependent manner (Fig. 2e). In parallel, ETC-168 treatment decreased cells of S and G2/M phases, while no significant induction of sub-G1 cells was observed in all three cells. On the contrary, ETC-168-insensitive LiSa-2 and LMS117 cells were in general unresponsive to the treatment. These data suggest that ETC-168 treatment inhibits cell proliferation and cell cycle progression mainly through a cytostatic effect. Based on IC_50_ values, LP6 and LPS141 represented ETC-168 sensitive LPS cells, while MESSA, SK-UT-1, and SK-UT-1B served as representative ETC-168 sensitive uterine LMS cells (Fig. 2f, g). LiSa-2 and LMS117 were considered as negative control lines whose IC_50_ values of ETC-168 were over 30 μM (Fig. 2f, g).

### MNK1/2 enforce eIF4E phosphorylation but have a limited impact on translational regulation

**Fig. 3:**
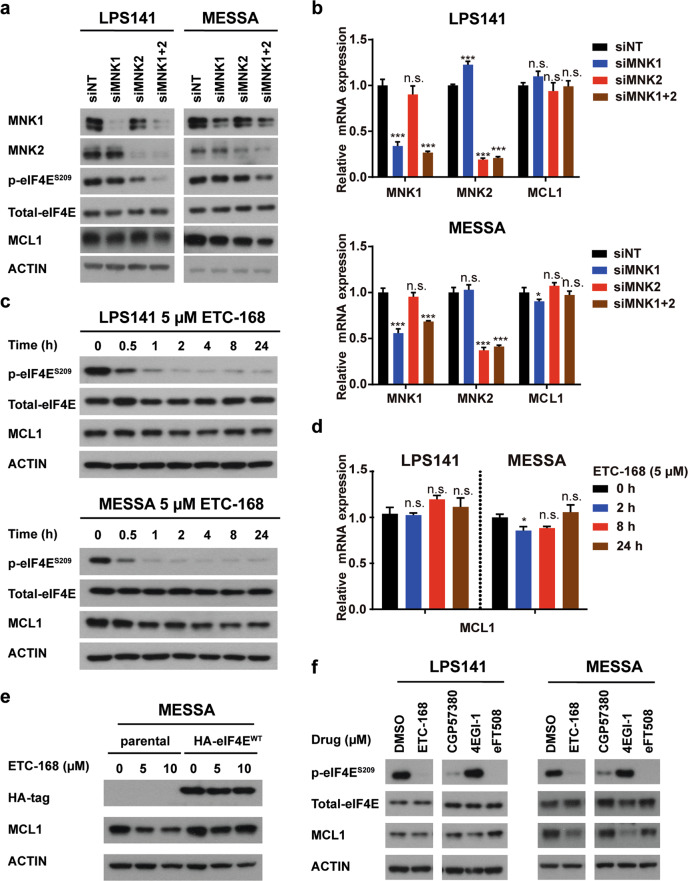
MNK1/2 and eIF4E regulate MCL1 expression in a subtype-specific manner. **a** Western blot showing effects of MNK1 and MNK2 silencing on eIF4E and MCL1 in LPS141 and MESSA cells. **b** qPCR analysis of mRNA expressions of MNK1, MNK2, and MCL1 in LPS141 and MESSA cells after siRNA treatment (48 h). **c** Western blot showing the expression of eIF4E and MCL1 in LPS141 and MESSA cells in response to ETC-168 treatment (5 μM). **d** Transcript level of MCL1 in LPS141 and MESSA cells in response to ETC-168 treatment (5 μM). **e** Expression of MCL1 in response to eIF4E overexpression and ETC-168 treatment (24 h). Lysates were derived from parental MESSA cells or MESSA cells stably expressing HA-tagged wild-type eIF4E. **f** Protein levels of eIF4E and MCL1 in LPS141 and MESSA cells upon treatment with ETC-168, CGP57380, 4EGI-1, and eFT508 (30 μM, 24 h). Data of **b**, **d** are shown as mean ± SD from representative data out of three independent experiments. Statistical significance is determined by either **b** two-tailed Student *t* test or **d** one-way ANOVA. n.s. not significant; *p < 0.05; **p < 0.01; ***p < 0.001.

As eIF4E is a well-documented substrate of MNK kinases, we reasoned that phosphorylation of eIF4E at Ser^209^ site (p-4E) may serve as an indirect readout of cellular responsiveness to MNK inhibition. Indeed, simultaneous silencing of MNK1 and MNK2 by siRNAs potently suppressed p-4E, while single knockdown of either MNK1 or MNK2 showed limited impact (Fig. 3a). Hence, MNK1 and MNK2 have a synergistic function to phosphorylate eIF4E in STS cells. Interestingly, inhibition of MNK1/2 by ETC-168 elevated the expression of MNK1/2 at both transcript and protein levels, suggestive of a potential feedback mechanism via which STS cells turn on a compensatory gene expression program upon kinase inhibition (Supplementary Fig. S2b, c).

MCL1, an anti-apoptosis gene [27], has been previously reported as a translational target of MNK1/2 and p-4E [28]. In our study, we found that MCL1 was responsive to MNK1/2 inhibition in a subtype-specific manner. Either co-depletion of MNK1/2 or ETC-168 treatment decreased MCL1 protein in uterine LMS cells without any reduction in either MCL1 transcripts or protein half-life (Fig. 3a, b and Supplementary Fig. S3a, b). However, neither protein nor mRNA level of MCL1 responded to MNK1/2 knockdown in LPS141 cells (Fig. 3a, b). Mirroring the phenotypes of genetic silencing, ETC-168 abolished p-4E in both LPS141 and MESSA within 1 h, whereas it reduced MCL1 protein only in MESSA cells independent of transcription (Fig. 3c, d). Ectopic overexpression of eIF4E in MESSA cells can counteract the inhibitory effect of ETC-168 on MCL1 (Fig. 3e).

Comparative study of three MNK kinase inhibitors (ETC-168, CGP57380, and eFT508) indicated a uniform inhibition of p-4E at 30 μM. 4EGI-1, an inhibitor of eIF4E-eIF4G interaction, did not have this potency (Fig. 3f). In terms of p-4E inhibition, ETC-168 and eFT508 showed a comparable activity, while CGP57380 was less proficient (Figs. 2b and 3f). As a subtype-specific translational target of MNK1/2, MCL1 protein declined upon treatment with ETC-168, eFT508, and 4EGI-1 in MESSA cells but not in LPS141 cells (Fig. 3f and Supplementary Fig. S3c). Notably, albeit p-4E has been shown as a key factor to enhance oncogenic translation function by MNK1/2, cellular responsiveness of p-4E to MNK kinase inhibitors decoupled from the growth-inhibitory effect of these compounds in STS. At the dose of 30 μM, ETC-168 effectively suppressed both p-4E and cell viability, whereas CGP57380 and eFT508 showed minor impact on cell viability in spite of p-4E inhibition (Figs. 2c and 3f). Together, these results suggest involvement of alternative mechanism other than p-4E-dependent translation in mediating the STS-promoting activity of MNK1/2.

### ETC-168 suppresses phosphorylation of ribosomal protein S6 (RPS6) in sensitive STS cells

To explore impact of ETC-168 on intracellular signalling transduction, we performed a slide-based phospho-protein array. Treatment of LPS141 cells with ETC-168 (10 μM, 8 h) showed a sharp inhibition of p-RPS6 at Ser^235/236^ site which is a well-recognized readout of mTOR activity (Supplementary Fig. S4a). This finding was verified by western blot analysis in various LPS and LMS cell lines (Supplementary Fig. S4b). Both p-RPS6 and its upstream kinases (S6K: p70S6K and p85S6K) were selectively downregulated by ETC-168 in a dose-dependent manner in all sensitive STS cells but stayed resistant in LMS117 and LiSa-2 cells (Supplementary Fig. S4b, c). In contrast, p-4E was consistently inhibited by ETC-168 across all tested LPS and ULMS cells (Supplementary Fig. S4b). Therefore, reduced phosphorylation of RPS6 serves as a reliable biomarker associated with cellular response to MNK inhibitor ETC-168.

### E2F1, FOXM1, and WEE1 are sensitive to MNK1/2 inhibition in STS

**Fig. 4:**
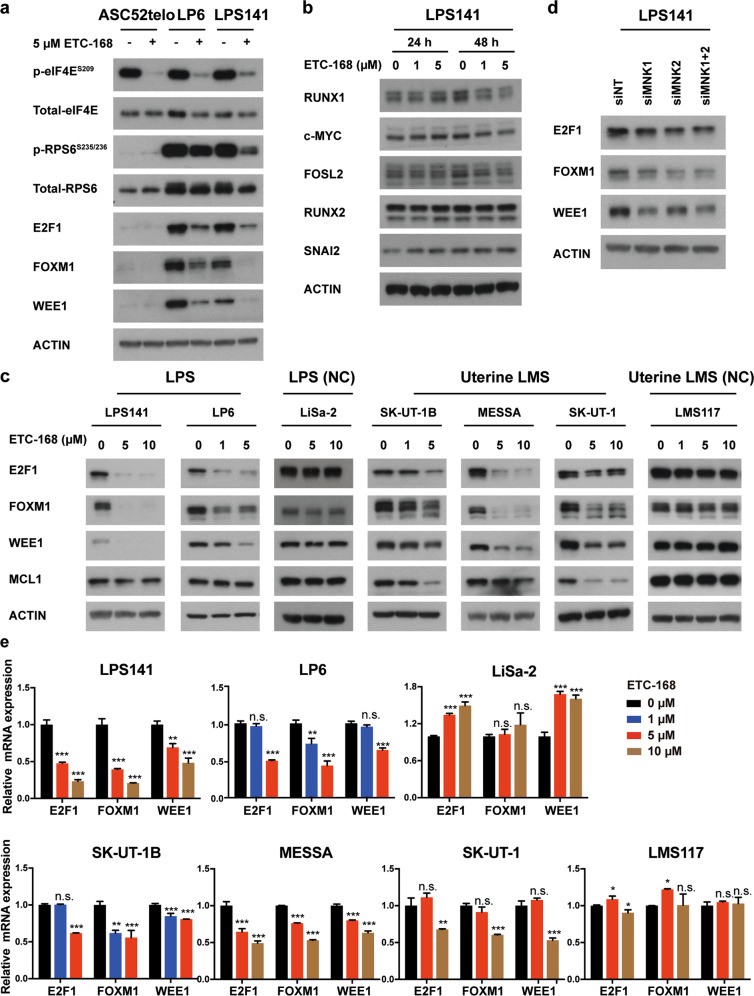
Inhibition of MNK1/2 decreases expression of E2F1, FOXM1, and WEE1. **a** Differential expression of eIF4E, RPS6, FOXM1, E2F1, and WEE1 in ASC52telo and de-differentiated LPS cells in either the presence or absence of ETC-168 (24 h). **b** Effect of ETC-168 on RUNX1, c-MYC, FOSL2, RUNX2 and SNAI2 proteins in LPS141 cells. **c** Effect of ETC-168 (24 h) on E2F1, FOXM1, WEE1, and MCL1 protein in STS cells. NC negative control cells. **d** Effect of siRNA-mediated silencing of MNK1 and MNK2 on E2F1, FOXM1, and WEE1 in LPS141 cells. **e** Response of E2F1, FOXM1, and WEE1 transcripts to ETC-168 treatment (24 h). Black, blue, red, and brown bars represent ETC-168 dosages of 0, 1, 5, and 10 μM, respectively. Data of **e** are shown as mean ± SD from representative data out of three independent experiments. Statistical significance is determined by one-way ANOVA. n.s. not significant; *p < 0.05; **p < 0.01; ***p < 0.001.

The surprising decoupling of p-4E inhibition from ETC-168-induced growth suppression in STS cells promoted us to explore alternative activity of MNK1/2 independent of translational regulation. Notably, along with our study, we found that expression of p-RPS6, E2F1, FOXM1, and WEE1 protein was elevated significantly in de-differentiated LPS cancer cells, relative to nonmalignant ASC52telo cells (Fig. 4a). Remarkably, tumor-specific expression of these proteins can be effectively blocked by ETC-168 treatment. Further analysis of ETC-168 treatment revealed a marked time-dependent reduction in protein/mRNA of E2F1, FOXM1, and WEE1 in both LPS141 and MESSA cells (Supplementary Fig. S5a, b). In addition, we examined protein levels of several LPS141 tumor-promoting genes (i.e., RUNX1, c-MYC, FOSL2, RUNX2, and SNAI2) after ETC-168 treatment [29]. However, these proteins showed either no or marginal response to ETC-168 (Fig. 4b). In contrast, protein levels of E2F1, FOXM1, and WEE1 were downregulated consistently by ETC-168 in drug-sensitive STS cells after 24-h drug incubation (Fig. 4a, c). Silencing of MNK1 and MNK2 also cooperatively decreased the abundance of E2F1, FOXM1, and WEE1 proteins in LPS141 cells (Fig. 4d). Of note, ETC-168 treatment also reduced mRNA levels of E2F1, FOXM1, and WEE1 in a dose-dependent manner in ETC-168-sensitive cells (Fig. 4e), while neither protein nor mRNA expression of these targets was downregulated by ETC-168 in LiSa-2 and LMS117 cells. MCL1 mRNA expression remained unchanged in all above treatments (Supplementary Fig. S5c).

**Fig. 5:**
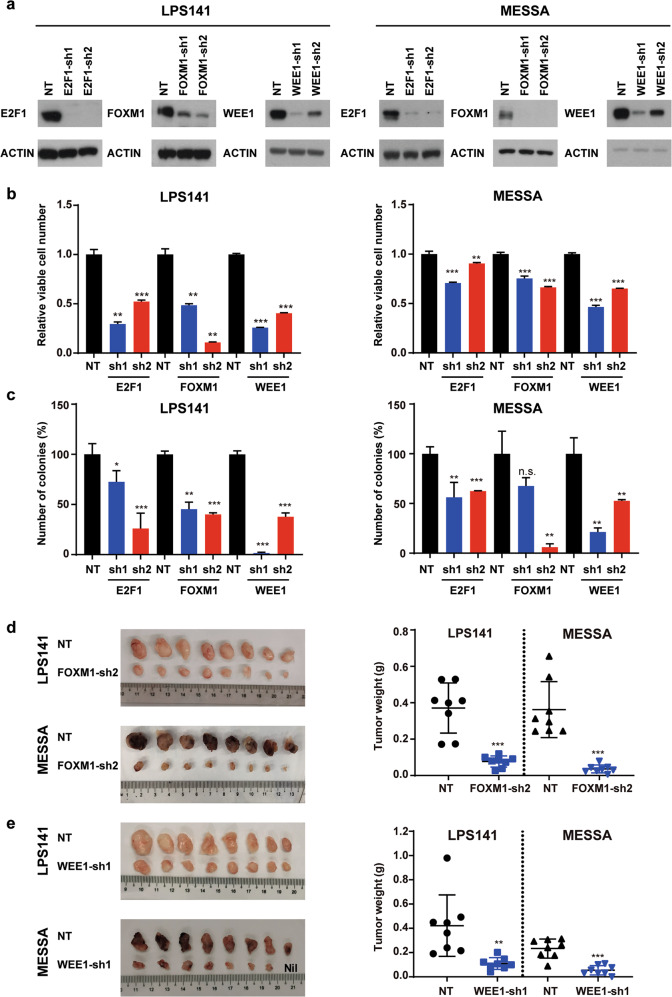
E2F1, FOXM1, and WEE1 are critical for soft-tissue sarcoma growth. **a** Verification of shRNA-mediated silencing of E2F1, FOXM1, and WEE1 in LPS141 and MESSA cells. **b**, **c** Effect of shRNA-mediated silencing of E2F1, FOXM1, and WEE1 on: **b** viability and **c** colony-forming ability of LPS141 and MESSA cells. **d**, **e** Effects of shRNA-mediated silencing of FOXM1 (*n* = 8 for each cell line) and WEE1 (*n* = 8 for each cell line) in LPS141 and MESSA cells on their tumorigenic ability in immunodeficient mice. Nil, no tumor growth. Data of **b** and **c** are shown as mean ± SD from representative data out of three independent experiments. Statistical significance is determined by two-tailed Student *t* test. n.s. not significant; *p < 0.05; **p < 0.01; ***p < 0.001.

FOXM1 [30], E2F1 [31], and WEE1 [32] have been implicated in the pathogenesis of human cancers, yet their roles remain vague in STS. We next examined consequence of genetic silencing of these genes in STS cells. Echoing the observations from MNK1/2 knockdown (Fig. 1), depletion of FOXM1, E2F1, and WEE1 attenuated the cell viability and anchorage-independent growth of both LPS141 and MESSA cells (Fig. 5a–c). Importantly, silencing of FOXM1 and WEE1 also impaired subcutaneous tumor formation by STS cells (Fig. 5d, e). Together, these results support that MNK1/2 sustain the expression of FOXM1, E2F1, and WEE1 to promote STS.

### MCL1 is a targetable dependency whose inhibition potentiates anti-STS efficacy of ETC-168

**Fig. 6:**
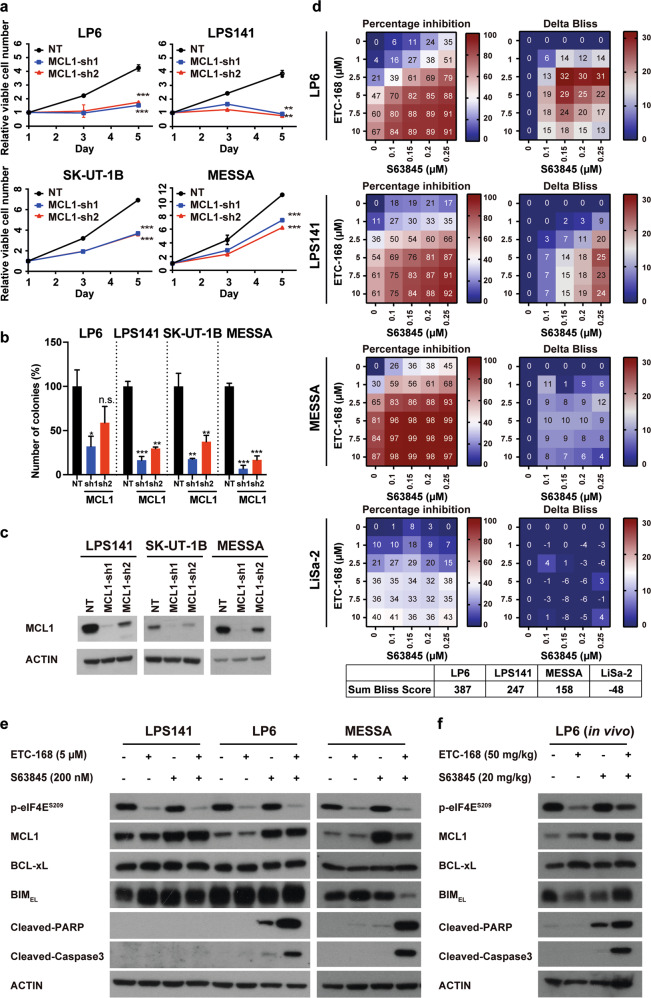
Inhibition of MCL1 synergizes with ETC-168 against STS cells. Effect of MCL1 ablation on: **a** viability and **b** anchorage-independent growth of STS cells. **c** Verification of shRNA-mediated silencing of MCL1. **d** Drug matrix heatmap showing the inhibitory impact and Delta Bliss Score upon ETC-168 treatment in combination with S63845 in LP6, LPS141, MESSA, and LiSa-2 cells. Representative matrix heatmap grid was showed from three independent experiments. **e**, **f** Western blot showing the effect of ETC-168 and S63845 (24 h) on expression of eIF4E and proteins related to apoptosis in **e** STS cells in vitro and **f** LP6 tumor grafts in vivo. Data of **a** and **b** are shown as mean ± SD from representative data out of three independent experiments. Statistical significance is determined by two-tailed Student *t* test. n.s. not significant; *p < 0.05; **p < 0.01; ***p < 0.001.

Uterine LMS cells were in general more sensitive to ETC-168 than LPS cells (Fig. 2f, g). Together with the observation that MCL1 is a uterine LMS-specific downstream target of MNK1/2, we hypothesized that reduced MCL1 expression can synergize with other effects from MNK inhibition to inhibit STS. To test this, we first examined the functional importance of MCL1 in STS. Genetic knockdown experiment demonstrated that MCL1 was essential for the viability and clonogenicity of both LPS (LP6, LPS141) and uterine LMS (SK-UT-1B, MESSA) cells (Fig. 6a–c). Remarkably, pharmacological inhibition of MCL1 by S63845, a small molecule with high binding affinity to the BH3-binding groove of MCL1 [33], potently inhibited the viability of MESSA, LPS141, and LP6 cells with IC_50_ values of 0.96, 0.25, and 0.26 μM, respectively (Supplementary Fig. 6a). These data suggest that MCL1 is a targetable growth dependency of STS cells. Next, we explored the combinational potential of MCL1 inhibitor and MNK inhibitor based on Bliss model [34]. In de-differentiated LPS cells, S63845 and ETC-168 exhibited a strong, synergistic growth-inhibitory effect in vitro, as evidenced by a Sum Bliss Score above 240 in both LPS141 and LP6 cells (Fig. 6d). Additionally, in uterine LMS cells MESSA, simultaneous inhibition of MCL1 and MNK1/2 achieved synergism in inhibition of cell viability (Fig. 6d). Co-treatment of S63845 and ETC-168 induced pronounced cleavage of Poly (ADP-Ribose) Polymerase 1 (PARP1) and Caspase 3 in LPS141, LP6, and MESSA cells, indicative of an enhanced apoptotic cell death (Fig. 6e and Supplementary Fig. 6b). However, no synergy of drug combo was observed in ETC-168-insensitive LiSa-2 cells (Fig. 6d). Hence, inhibition of MCL1 augments the efficacy of ETC-168 in MNK1/2 inhibitor-responsive STS cells in vitro. To explore activity of ETC-168 and S63845 in vivo, mice bearing LPS xenografts were randomized into four groups for drug treatment: (1) vehicle control; (2) ETC-168 treatment (50 mg/kg, p.o., b.i.d.); (3) S63845 treatment (20 mg/kg, i.p., q.d.); (4) Combination of ETC-168 and S63845 treatment (Supplementary Fig. 6c). Echoing our observations made from in vitro treatment, ETC-168 treatment strongly inhibited p-4E in tumor grafts, while S63845 treatment stabilized MCL1 (Fig. 6f). Single agent treatment with S63845 triggered a modest induction of cleaved-PARP, while combination of ETC-168 and S63845 exerted a robust synergy in activating the expression of both cleaved-PARP and cleaved-Caspase 3. These data indicate a promising bioavailability and in vivo activity of ETC-168/S63845 combo in tumor-bearing mice, providing valuable insights for follow-up studies of developing optimal delivery strategy, as well as improving dosing and schedule of drug treatment.

## Discussion

**Fig. 7:**
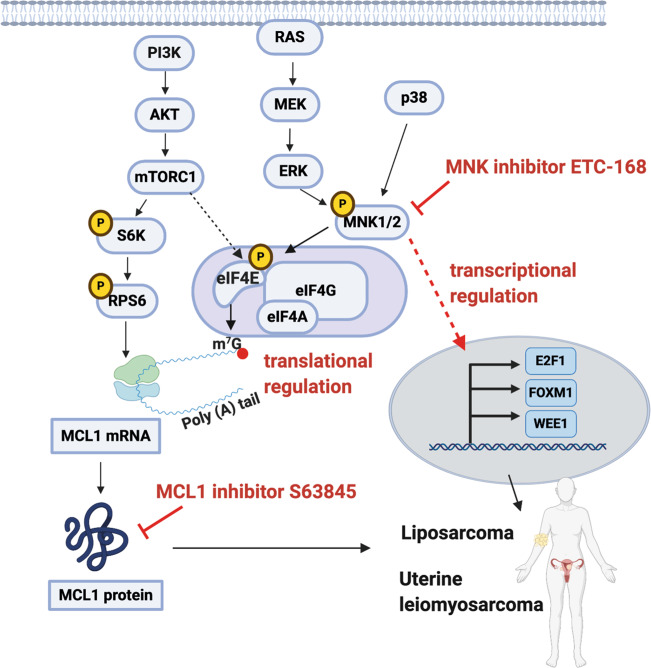
Graphical summary of MNK1/2 function in STS and rationale for co-targeting MNK1/2 and MCL1. MNK inhibitor ETC-168 is able to disrupt both phospho-eIF4E axis and MNK1/2-dependent transcriptional regulation of E2F1, FOXM1, and WEE1. Blockage of MCL1 activity synergizes with MNK1/2 inhibition against STS cells.

In this study, we provide the rationale and strategy for targeting MNK1/2 in STS. Both MNK1/2 and their newly identified downstream targets including E2F1, FOXM1, and WEE1 enforce oncogenic potentials of STS cells. We demonstrate that a novel, potent MNK inhibitor ETC-168 can effectively block p-4E and transcription of E2F1, FOXM1, and WEE1, along with its antiproliferative activity in STS cells. We further uncover p-RPS6 as a promising biomarker associated with ETC-168 responsiveness, and concordant inhibition of MCL1 as a favorable strategy for drug combo (Fig. 7). In line with previous reports [23, 35–37], our data support the role of MNK1 and MNK2 as oncogenes and therapeutic targets. Both MNK1 and MNK2 proteins were elevated in many STS cell lines when compared with nonmalignant controls. Activities of MNK1/2 are correlated positively with their expression levels, as suggested by the inhibitory effect of MNK1/2 silencing on p-4E (Fig. 3a). Of note, upregulation of phospho-MNK1/2 was also prevalent among STS cells. Since ERK1/2 and p38 have been shown as the major upstream regulators of phospho-MNK1/2 [38, 39], elevation of phospho-MNK1/2 in sarcoma cells is likely resulted from hyper-activation of mitogen-activated protein kinases in cancerous state.

Interestingly, MNK2 showed a higher degree of overexpression than MNK1. MNK2 also exerted stronger regulatory effects on both p-4E and in vivo tumor expansion of STS cells. Hence, MNK2 may play a dominant role to maintain the steady level of p-4E in STS cells and to enforce their tumorigenic potentials. This finding was consistent with the notion that MNK1 behaves as a stress-responsive kinase with a low basal activity while MNK2 has a higher basal activity [9]. However, our data suggest that MNK1 silencing alone is insufficient to block p-4E in STS cells, which is different from previous observations that either MNK1 inhibitor or MNK1 depletion suppressed the expression of p-4E [13, 35]. Instead, simultaneous targeting of MNK1 and MNK2 shows synergistic effect on p-4E. Importantly, Mnk1/2 double knockout mice are viable and compatible with normal development [40], while Mnk1/2-deficient cells are refractory to oncogenic transformation. Since loss of MNK1/2 can be tolerated by normal cells, MNK1/2 are desirable cancer targets for pharmacologic inhibition.

Strong genetic dependence of STS cells on MNK1/2 establishes the rationales for MNK-targeted therapy. Several small-molecule inhibitors against MNK1/2 kinase activity have been developed including CGP57380, eFT508, BAY1143269, and SEL201. In this study, we report the anti-STS activity of a newly developed dual MNK1/2 inhibitor ETC-168. Our comparative evaluation of ETC-168, CGP57380, and eFT508 revealed a superior antiproliferative efficacy of ETC-168 against STS cells. Both ETC-168 and eFT508 showed better capability to inhibit p-4E than CGP57380. Reduction of p-4E was observed within 30 min of treatment with ETC-168, suggestive of a rapid target engagement. However, to our surprise, growth-inhibitory effect of these compounds does not correlate with their impact on p-4E. Inhibition of MNK1/2 uniformly reduced p-4E in MNK1/2 inhibitor-insensitive cells. These data implicate additional p-4E-independent mechanism in growth suppression upon MNK1/2 inhibition. Besides, our findings suggest that p-4E is dispensable for STS cell viability.

As a known target gene of MNK1/2-eIF4E, MCL1 responded to MNK1/2 inhibitors only in uterine LMS cells. Inhibition of MNK1/2-eIF4E showed a limited impact on protein synthesis of MCL1 in LPS cells. Remarkably, cells retaining MNK1/2-dependent expression of MCL1 were more sensitive to MNK1/2 inhibition, suggesting that MCL1 inhibition synergizes with other growth-inhibitory effects from MNK1/2 inhibitors. In support of this notion, MCL1 inhibitor showed an encouraging activity to augment the efficacy of ETC-168. The differential kinetics and strength of cleaved-PARP and cleaved-Caspase 3 between LP6 and LPS141 in response to ETC-168/S63845 combo (Fig. 6e and Supplementary Fig. 6b) may be associated with cell line-specific genetic backgrounds (e.g., steady levels of anti-apoptotic proteins). Combination of MCL1 inhibitor and MNK1/2 inhibitor is a promising strategy for further therapeutic development.

We identify p-RPS6 as a responsive biomarker for MNK1/2 inhibitor ETC-168. As a downstream target of RPS6 kinases (p70S6K and p85S6K) and mTOR, p-RPS6 has been shown as a candidate biomarker to predict cellular response to mTOR inhibitor [41]. Along with a reduction in p-RPS6, combined application of MNK1/2 inhibitor and mTOR inhibitor has shown synergism against glioma growth [35]. Phospho-S6K levels were markedly downregulated by ETC-168 in drug-sensitive cells (LPS141, LP6, SK-UT-1B, MESSA, and SK-UT-1), while both p70S6K and p85S6K were less responsive to ETC-168 in drug-insensitive cells (LiSa-2 and LMS117). These data suggest that S6K inhibition may partially contribute to the antiproliferative activity of ETC-168. So far, it remains elusive how ETC-168 inhibits p-RPS6 in STS cells. Nevertheless, the strong and selective inhibition of p-RPS6 by ETC-168 suggests that S6K activity can be effectively compromised upon ETC-168 treatment in responsive STS cells. In support of this observation, MNK1/2 also act as inhibitors of DEPTOR-mTORC1 binding and stabilizers of TELO2-mTORC1, thereby enhancing mTORC1 function [19]. Additionally, MNK1/2 have been reported to regulate S6K in a kinase-independent manner [42]. It is possible that ETC-168 impairs p-RPS6 via either direct inhibition of MNK1/2-mTOR-S6K signalling components or disruption of MNK1/2 substrates which indirectly regulate RPS6 phosphorylation in STS cells. Inhibition of p-RPS6 may synergize with that of p-4E in suppression of STS proliferation. In addition to p-RPS6, basal expression of MNK2 serves as another potential predictive marker for ETC-168. Sarcoma cells with high expression of both MNK1 and MNK2 (e.g., LPS141, LP6, MLS402, MESSA, SK-UT-1, SK-UT-1B, and T778) were more responsive to MNK inhibitors (Fig. 2c). Most of ETC-168-insensitive cells expressed a smaller amount of MNK2 proteins, e.g., ASC52telo, LiSa-2, and LMS117. Further increase in sample size will strengthen this observation.

Apart from p-4E-dependent translation, MNK1/2 have been shown to regulate transcription of the oncogenic ANGPTL4 [20]. We also uncover that MNK1/2 maintain active transcription of three crucial STS-promoting genes, i.e., FOXM1, E2F1, and WEE1. Notably, all three genes are well known as drivers of mitotic cell cycle [43]. ETC-168 demonstrated a robust suppression of all three genes in responsive cells, which was accompanied by a marked disruption of cell cycle progression. A systematic examination of ETC-168-responsive transcriptome may help to discover key transcriptional nodules/modulators mediating the activity of MNK1/2. As our phospho-protein array indicated a clean and specific activity of ETC-168 on intracellular signalling transduction, it is unlikely that MNK1/2 regulate gene transcription via signalling crosstalk. Alternatively, MNK1/2 may participate in gene transcription by regulating transcription apparatus, nuclear transport (e.g., nucleoporins and karyopherins [44, 45]) or transcription factors (e.g., peroxisome proliferator-activated receptors [46]). ETC-168 can be used as a useful compound and surrogate tool to block oncogenic expression of E2F1, FOXM1, and WEE1 in human cancers. ETC-168 may be useful for drug combination, especially for cases that genetic inhibition of these genes has been proven synergistic with other therapeutic agents. As both FOXM1 and E2F1 are master transcription factors in human cancer, inhibition of MNK1/2-dependent expression of FOXM1 and E2F1 will have a broad impact on transcriptome.

Collectively, this study reveals a druggable genetic dependency of STS cells on MNK1/2 and their novel downstream targets including E2F1, FOXM1, and WEE1. Pharmacologic inhibition of MNK1/2 can impair both expression of transcriptional targets and p-4E-dependent oncogenic translation. Therapeutic targeting of MNK proteins holds the promise for STS treatment.

## Materials and methods

### Cell culture

Human embryonic kidney cell line HEK293T, LMS cell lines LMS1, LMS117, SK-UT-1, and SK-UT-1B were maintained in Dulbecco’s Modified Eagle Medium (DMEM, Biowest). SK-LMS-1 cells were cultured in Minimum Essential Media (MEM, Gibco). LMS cell line MESSA was maintained in McCoy’s 5 A (Modified) Medium (McCoy, Gibco^TM^). Adipose mesenchymal stem cell ASC52telo was cultured in mesenchymal stem cell basal medium supplemented with mesenchymal stem cell growth Kit (ATCC). Human primary uterine smooth muscle cell (HUtSMC) was maintained in vascular cell basal medium supplemented with vascular smooth muscle cell growth kit (ATCC). Shef-DDLPS01, Shef-DDLPS02, Shef-LMS01-w1, and Shef-LMS01-ws cells were kindly provided by Drs Karen Sisley and Abdulazeez Salawu [47]. RH5 was a kind gift from Dr Peter Houghton. LP6 and LPS141 cells were provided by Dr Christopher DM Fletcher [48]. MLS402, MLS-1765, and GOT3 cells were generously provided by Dr Pierre Åman [49–51]. T778 and T1000 cells were gifts from Dr Florence Pedeutour. 402-91/ET [52], FU-DDLS-1 [53], and LiSa-2 [54] cells were gifts from Dr Eugenio Erba, Dr Jun Nishio, and Dr Peter Möller, respectively. MESSA, SK-UT-1, and SK-UT-1B cells were kindly provided by Dr Stefan Fröhling. LMS117 and LMS1 were provided by Dr Raphael Pollock [55]. SK-LMS-1 was generously provided by Omid Khorram. LMS cell lines (Shef-LMS01-w1, Shef-LMS01-ws), alveolar RMS cell line RH5 and all LPS cell lines were maintained in Roswell Park Memorial Institute medium 1640 (RPMI-1640, Biowest). All of the above-mentioned cancer cell lines were cultured in respective media supplemented with 10% fetal bovine serum (FBS, Biowest) and 1% penicillin-streptomycin (Gibco). Cells were grown in a humidified incubator with 5% CO_2_ at 37 °C. All LPS cell lines have been authenticated by STR analysis with the Geneprint 10 System Kit (Promega) and tested negative for mycoplasma contamination.

### Plasmids and chemicals

All shRNA vectors (Supplementary Table S1) were cloned into pLKO.1 lentiviral vector. SHC002 was used as non-targeting control (NT). Either puromycin (Sigma-Aldrich) or blasticidin (Life Technologies) was used to select stable knockdown cell lines. pHAGE-Flag-HA-eIF4E-WT plasmid was a gift from Rebecca Lock [56]. siRNAs targeting MNK1 and MNK2 were purchased from Dharmacon. RNAiMAX (Life Technologies) was used to transfect siRNAs. ETC-168 was kindly provided by Experimental Drug Development Centre (EDDC), A*STAR, Singapore; CGP57380, eFT508, 4EGI-1, Cycloheximide, and S63845 were purchased from Abcam, MedChemExpress, Tocris Bioscience, Sigma-Aldrich, and Chemietek, respectively.

### Cell viability assay

Cell viability was detected by MTT (3-(4,5-Dimethylthiazol-2-yl)-2,5-diphenyltetrazolium bromide) assay. Briefly, STS cells were seeded in 96-well plates with 2000–3000 cells/well and then cultured for 72 h under indicated treatments. At the end of experiment, 10 µL MTT substrate (Sigma-Aldrich) was added into each well followed by 3-h incubation. MTT substrate was then removed. Cells were lysed by addition of 100 µL MTT stop solution. Absorbance at 570 nm was measured using a Tecan microplate reader.

### Soft agar colony formation assay

Soft agar colony formation assay was used to evaluate the anchorage-independent growth of STS cells. In brief, in each assay well of a 12-well plate, bottom layer was made by 500 µL of DMEM or RPMI medium containing 10% FBS and 0.5% agarose. Top layer was made by mixture of 1000–5000 STS cells and 500 µL of culture medium containing 10% FBS and 0.4% low melting agarose. Plates were put into a 4 °C freezer for 25 min followed by addition of 2 mL/well of complete medium. Plates were kept in a humidified incubator with 5% CO_2_ at 37 °C. After two to three weeks, colonies were stained with 0.01% crystal violet in 4% paraformaldehyde, and photographed using a dissection microscope.

### Xenograft assay

All animal experiments were conducted in accordance with ethical approval from the NUS Institutional Animal Care and Use Committee. In vivo tumorigenicity of genetically modified STS cells was accessed by subcutaneous xenograft assay. NSG mice (6- to 8-week-old) were used in this study. Indicated number of cells were mixed with 100 µL of PBS/Matrigel (BD Biosciences) solution (1:1) and subsequently injected subcutaneously into dorsal flank of recipient mice. For in vivo drug treatment, mice bearing palpable LP6 xenografts were randomized into four groups after one-week implantation. ETC-168 was given by oral gavage at a dose of 50 mg/kg (100 µL, Ora-Plus Oral Suspending Vehicle, Paddock Laboratories), twice a day for 2 days. S63845 was given by tail vein injection at a dose of 20 mg/kg (100 µL, 5% Solutol Vehicle), once a day for 2 days. Mice were sacrificed at the experimental endpoint for tumor harvest and subsequent protein extraction.

The sample sizes of mice in each experimental group were not based on statistical methods but on experience. The number of mice in each group had been described in the figure legends. No animals were excluded from analysis. No specific randomization and blinding methods were performed for all animal studies.

### Western blot assay

Cells were lysed with lysis buffer (50 mM Tris pH 8.0, 420 mM NaCl, 5% glycerol, 0.5% NP40, 1 mM EDTA) supplemented with 1 mM DTT, 1 mM phenylmethylsulfonyl fluoride (PMSF), 1 mM MgCl_2_, DNase (1:500, Thermo Fisher Scientific), 1× protease inhibitor cocktail (Roche) and 1× phosphatase inhibitor cocktail (Roche) for 30 min on ice. For detection of total and phospho-MNK1/2 proteins, cells were lysed by a modified lysis buffer (50 mM Tris pH 7.6, 150 mM NaCl, 0.2% NP-40, 2 mM EDTA) supplemented with 1 mM DTT, 1 mM PMSF, 1× protease inhibitor and 1× phosphatase inhibitor. Proteins from xenograft tumors were extracted by 1.2× RIPA buffer supplemented with 1 mM DTT, 1 mM PMSF, 1× protease inhibitor and 1× phosphatase inhibitor. Protein concentration was determined by Bradford assay. Western blot assay was carried out following a standard protocol. Quantification of western blot bands was done by ImageJ. Primary antibodies are listed in Supplementary Table S2.

### RNA preparation and real-time quantitative PCR (qPCR)

Total RNA was isolated using RNeasy Kit (Qiagen, 74106) according to the manufacturer’s instructions. Extracted RNA was treated with DNase and subjected to reverse transcription using RevertAid RT Reverse Transcription Kit (Thermo Fisher Scientific). qPCR was performed using Kapa SYBR fast qPCR Master Mix (KAPA Biosystems) on a 7500 Real-time PCR System (Applied Biosystems). qPCR primers are listed in Supplementary Table S3.

### Cell cycle analysis

STS cells were seeded in a 6 cm plate. After drug treatment for indicated concentrations and durations, cells were harvested and fixed in 70% ethanol at 4 °C overnight. Then cells were washed with PBS and resuspended in 40 mg/mL propidium iodide solution with RNase. Samples were incubated at 37 °C for 30 min in the dark, followed by DNA content analysis using LSR II Flow Cytometer System (BD Biosciences).

### Phospho-Kinase protein array

The Phospho-Kinase protein array was conducted using the PathScan^®^ RTK Signaling Antibody Array Kit (7949S, Cell Signaling Technology) according to the manufacturer’s instructions. LPS141 cells were treated with either DMSO or 10 μM ETC-168 for 8 h.

### Statistical analysis

Bliss model was used to quantify the combination effect of two drugs. “Bliss expectation” formula is *(A* + *B* *−* *A* *×* *B)*, where *A* and *B* represent the growth inhibition of drug A and B at a given dose. “Delta Bliss” is the difference between real growth inhibition and Bliss expectation of the combination of drug A and B at the same dose. “Sum Bliss Score” is the sum of Delta Bliss across whole dose matrix. Statistical analyses were performed using GraphPad Prism. The data met the assumptions of the tests and all data were presented as mean ± SD. Statistical analysis was reported using either two-tailed Student’s *t* test or one-way ANOVA between control group and experimental group. n.s., not significant; *P < 0.05; **P < 0.01; ***P < 0.001.

## Supplementary information

supplementary
