# Supplementary material for: MNK1 and MNK2 enforce expression of E2F1, FOXM1, and WEE1 to drive soft tissue sarcoma
Source: Oncogene. 2021 Feb 9;40(10):1851–67. doi: 10.1038/s41388-021-01661-4 (PMC7946644; doi:10.1038/s41388-021-01661-4)
Supplement: Supplementary file 1 — supplementary [file 41388_2021_1661_MOESM1_ESM.docx]

**Supplementary Information**

**MNK1 and MNK2 enforce expression of E2F1, FOXM1 and WEE1 to drive**

**soft tissue sarcoma**

Xin-Yu Ke, Ye Chen, Valarie Yu-Yan Tham, Ruby Yu-Tong Lin, Pushkar​ ​Dakle, Kassoum Nacro, Mark Edward Puhaindran, Peter Houghton, Angela Pang, Victor Kwanmin Lee, Ling-Wen Ding, Sigal Gery, Jeffrey Hill, Leilei Chen, Liang Xu, H. Phillip Koeffler

• **SUPPLEMENTARY TABLES**

• **SUPPLEMENTARY FIGURES**

**SUPPLEMENTARY TABLES**

**Supplementary Table S1: Sequence of shRNAs**

| **Vector** | **Sequence (5’ -> 3’)** |
| --- | --- |
| MNK1-sh1 | CCGGGCTGCTAAAGTCAGTAGTATCCTCGAGGATACTACTGACTTTAGCAGCTTTTTG |
| MNK1-sh2 | CCGGGCCAGGAAAGTTTGAAGATATCTCGAGATATCTTCAAACTTTCCTGGCTTTTTG |
| MNK1-sh3 | CCGGCTTGCTCTTCTTTCTAGAATGCTCGAGCATTCTAGAAAGAAGAGCAAGTTTTTG |
| MNK2-sh1 | CCGGCCTGGGCGTCATCTTGTATATCTCGAGATATACAAGATGACGCCCAGGTTTTTG |
| MNK2-sh2 | CCGGCGCCGTCAAGATCATTGAGAACTCGAGTTCTCAATGATCTTGACGGCGTTTTTG |
| MNK2-sh3 | CCGGCCTAGAGCTGATTGAGTTCTTCTCGAGAAGAACTCAATCAGCTCTAGGTTTTTG |
| MNK2-sh4 | CCGGCATGTGTTAATGTTACGATGTCTCGAGACATCGTAACATTAACACATGTTTTTG |
| FOXM1-sh1 | CCGGTTGCAGGGTGGTCCGTGTAAACTCGAGTTTACACGGACCACCCTGCAATTTTTG |
| FOXM1-sh2 | CCGGAGGACCACTTTCCCTACTTTACTCGAGTAAAGTAGGGAAAGTGGTCCTTTTTTG |
| E2F1-sh1 | CCGGACCTCTTCGACTGTGACTTTGCTCGAGCAAAGTCACAGTCGAAGAGGTTTTTTG |
| E2F1-sh2 | CCGGGACCTCTTCGACTGTGACTTTCTCGAGAAAGTCACAGTCGAAGAGGTCTTTTTG |
| MCL1-sh1 | CCGGGCCTAGTTTATCACCAATAATCTCGAGATTATTGGTGATAAACTAGGCTTTTTG |
| MCL1-sh2 | CCGGGCTTCGGAAACTGGACATCAACTCGAGTTGATGTCCAGTTTCCGAAGCTTTTTG |
| WEE1-sh1 | CCGGCGCTCTGTCAGCCTTACTATACTCGAGTATAGTAAGGCTGACAGAGCGTTTTTG |
| WEE1-sh2 | CCGGATAAACCGATCTTCGTGATACCTCGAGGTATCACGAAGATCGGTTTATTTTTTG |

**Supplementary Table S2: List of primary antibodies**

| **Antibody** | **Company** | **Catalog** |
| --- | --- | --- |
| β-ACTIN | Sigma-Aldrich | A1978 |
| MNK1 | Cell signaling Technology | 2195 |
| MNK2 | Sigma-Aldrich | M0696 |
| pMNK1^Thr197/202^ | Cell signaling Technology | 2111S |
| pMNK1^Thr255^ | Signalway Antibody | 12142-1 |
| pMNK1^Thr385^ | Abcam | Ab138671 |
| pMNK2^Thr249^ | Signalway Antibody | 12517-1 |
| peIF4E^Ser209^ | Abcam | Ab76256 |
| eIF4E | Cell signaling Technology | 2067 |
| pRPS6^Ser235/236^ | Cell signaling Technology | 2211 |
| RPS6 | Cell signaling Technology | 2217 |
| pp70S6K^Thr421/Ser424^ | Cell signaling Technology | 9204 |
| P70S6K | Cell signaling Technology | 9202 |
| FOXM1 | Thermo Fisher Scientific | PA5-27631 |
| E2F1 | Cell signaling Technology | 3742 |
| MCL1 | Cell signaling Technology | 5453 |
| WEE1 | Cell signaling Technology | 13084 |
| BCL-xL | Santa Cruz | sc-136132 |
| BIM | Cell signaling Technology | 2933 |
| Cleaved-PARP | Cell signaling Technology | 5625 |
| Cleaved-Caspase3 | Cell signaling Technology | 9661 |
| RUNX1 | Santa Cruz | Sc-365644 |
| c-MYC | Cell signaling Technology | 5605 |
| FOSL2 | Cell signaling Technology | 19967 |
| RUNX2 | Cell signaling Technology | 8486 |
| SNAI2 | Cell signaling Technology | 9585 |

**Supplementary Table S3: List of qRT-PCR primers**

| **Primer** | **Sequence (5’ -> 3’)** |
| --- | --- |
| ACTIN-F | GACGACATGGAGAAAATCTG |
| ACTIN-R | ATGATCTGGGTCATCTTCTC |
| MNK1-F | GCTGACCTCTGAATTGCTTGG |
| MNK1-R | TCGATGATTTTGACGGCATACTC |
| MNK2-F | TCAAGATCATTGAGAAGCAG |
| MNK2-R | AAGAACTCAATCAGCTCTAGG |
| MCL1-F | GTGCCTTTGTGGCTAAACACT |
| MCL1-R | AGTCCCGTTTTGTCCTTACGA |
| FOXM1-F | TCTTTGTTTATCAGTGCTGC |
| FOXM1-R | TTTCTTCCTCCTTGATAGTCTG |
| E2F1-F | CTGATGAATATCTGTACTACGC |
| E2F1-R | CTTTGATCACCATAACCATCTG |
| WEE1-F | TCAATGGCATGAAATCAGAC |
| WEE1-R | CTGGATCTGGATGAATCATAAC |

**SUPPLEMENTARY FIGURES**

**
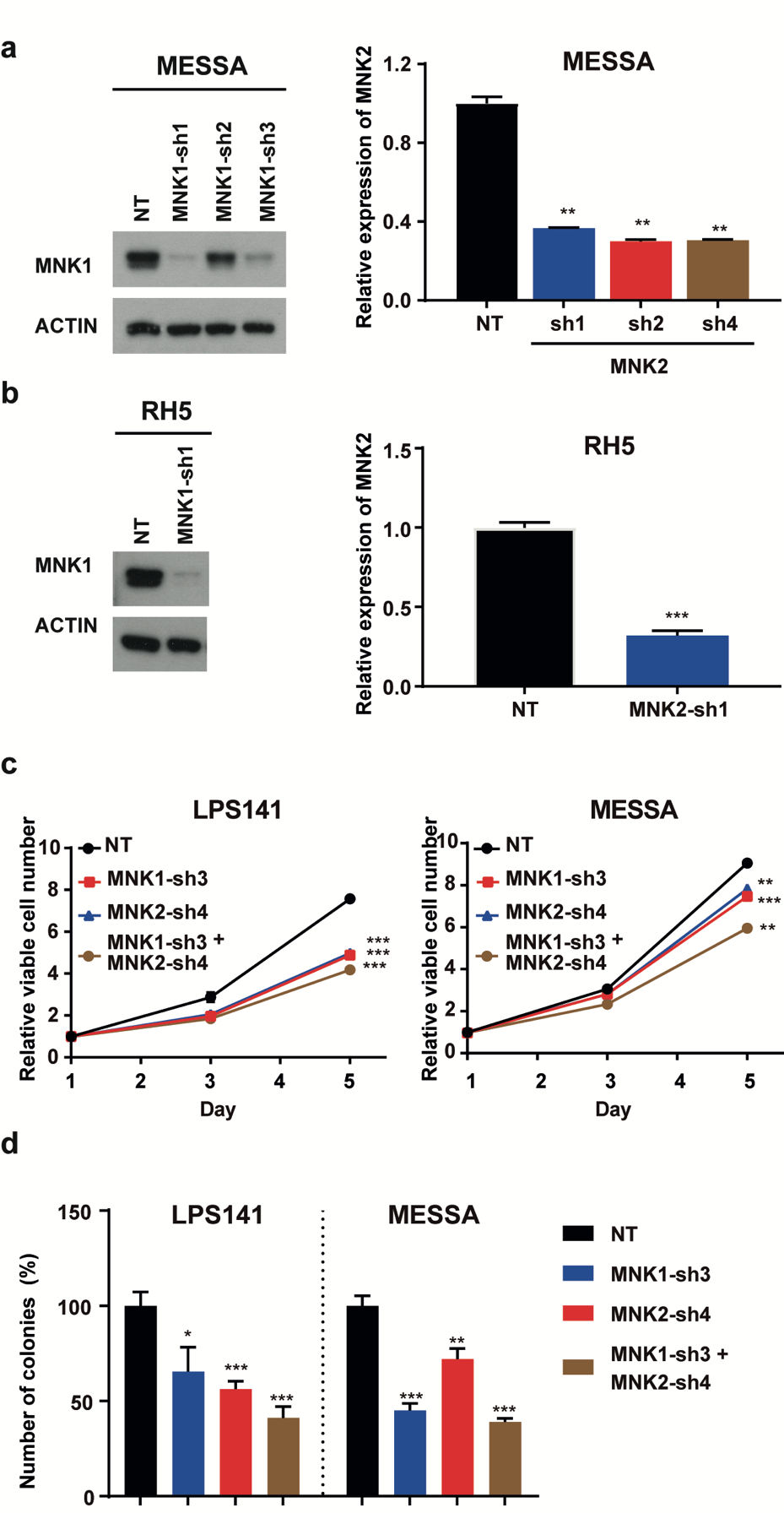
**

**Supplementary Figure S1.**

**Expression and function of MNK1 and MNK2 in STS cells.**

**a and b** Verification of shRNA-mediated knockdown of MNK1 and MNK2 as measured by either western blot or qPCR analysis in **(a)** MESSA and **(b)** RH5 cells. **c and d,** Effect of either single or double knockdown of MNK1/2 on **(c)** cell viability and **(d)** anchorage-independent growth of LPS141 and MESSA cells. Data are shown as mean ± SD from representative data out of three independent experiments. Statistical significance is determined by two-tailed Student *t*-test. n.s., not significant; *: p<0.05; **: p<0.01; ***: p<0.001.

**
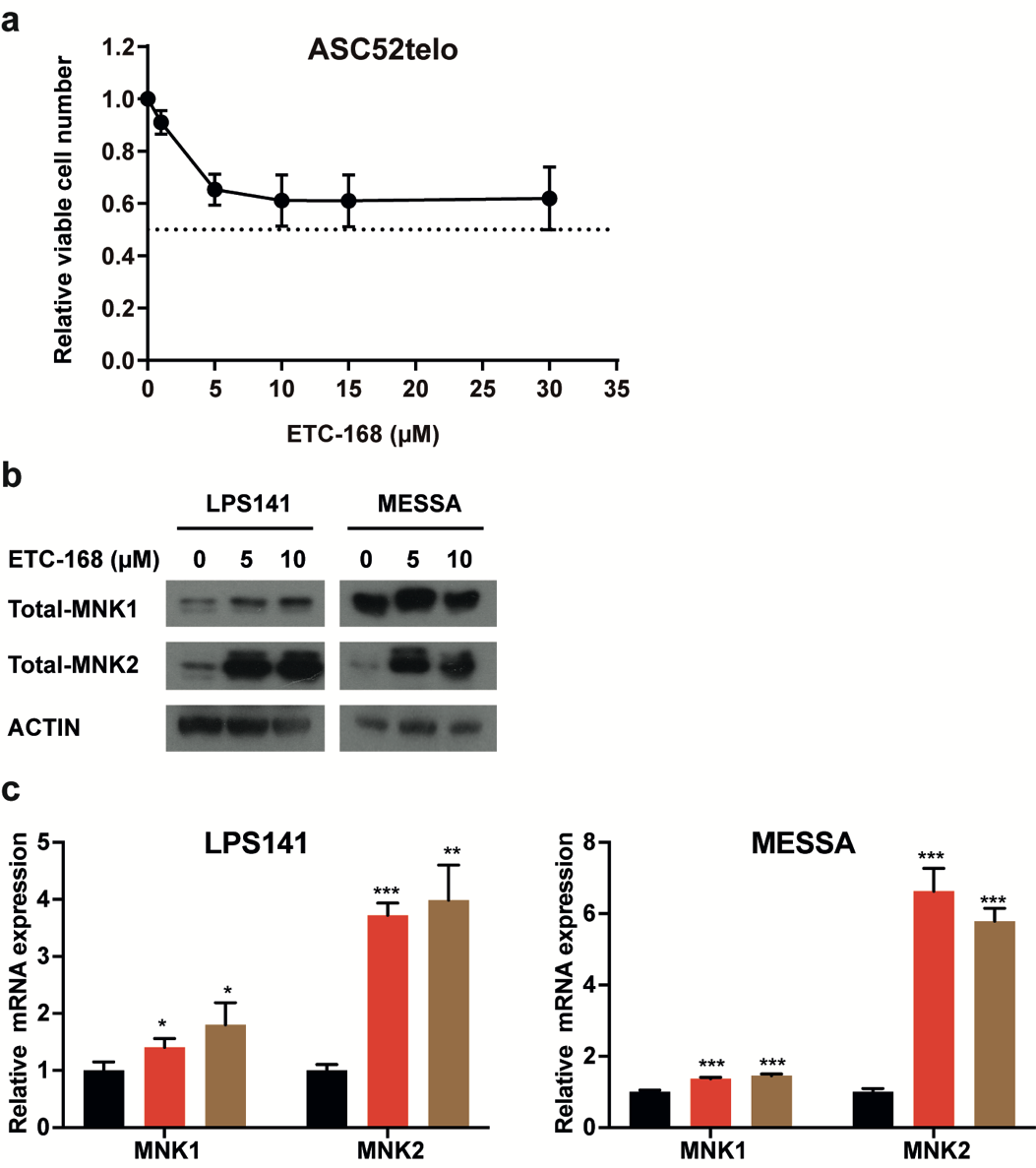
**

**Supplementary Figure S2.**

**Activity and function of ETC-168 on normal and STS cells.**

**a** Response of mesenchymal stem cells ASC52telo to ETC-168 treatment (72 hours). Experiment was conducted in biological triplicates. **b and c** Effect of ETC-168 on **(b)** protein and **(c)** mRNA expression of MNK1/2 in LPS141 and MESSA cells. Data of **a** and **c** represent mean ± SD (n=3). Statistical significance is determined by two-tailed Student *t*-test. n.s., not significant; *: p<0.05; **: p<0.01; ***: p<0.001.

**
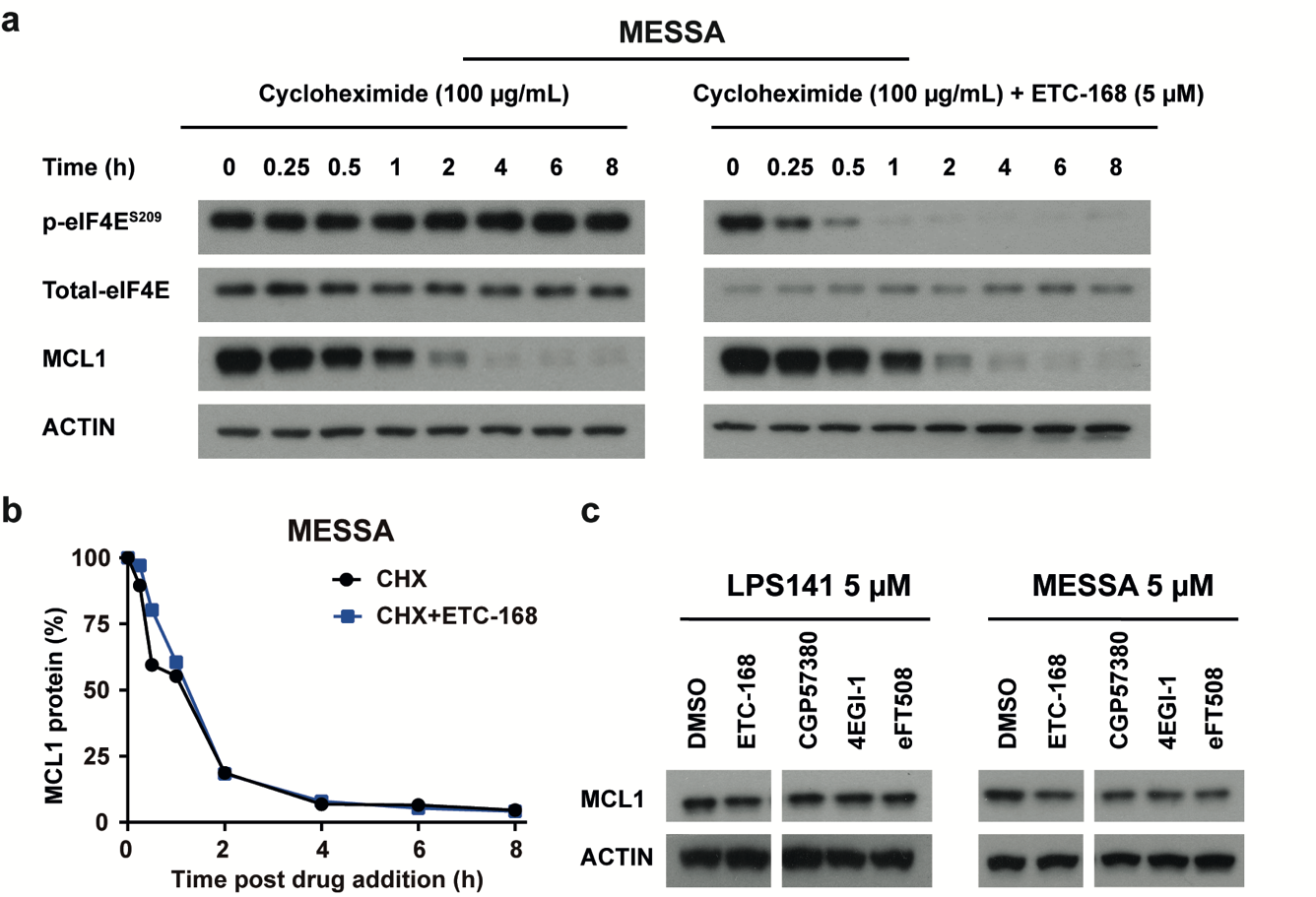
**

**Supplementary Figure S3.**

**ETC-168 regulates MCL1 protein expression without affecting its protein half-life.**

**a and b** Effect of ETC-168 on protein half-lives of MCL1 and eIF4E in MESSA cells. CHX, cycloheximide. **c** Western blot analysis of MCL1 expression in response to MNK/eIF4E inhibitors (24 hours) in LPS141 and MESSA cells.


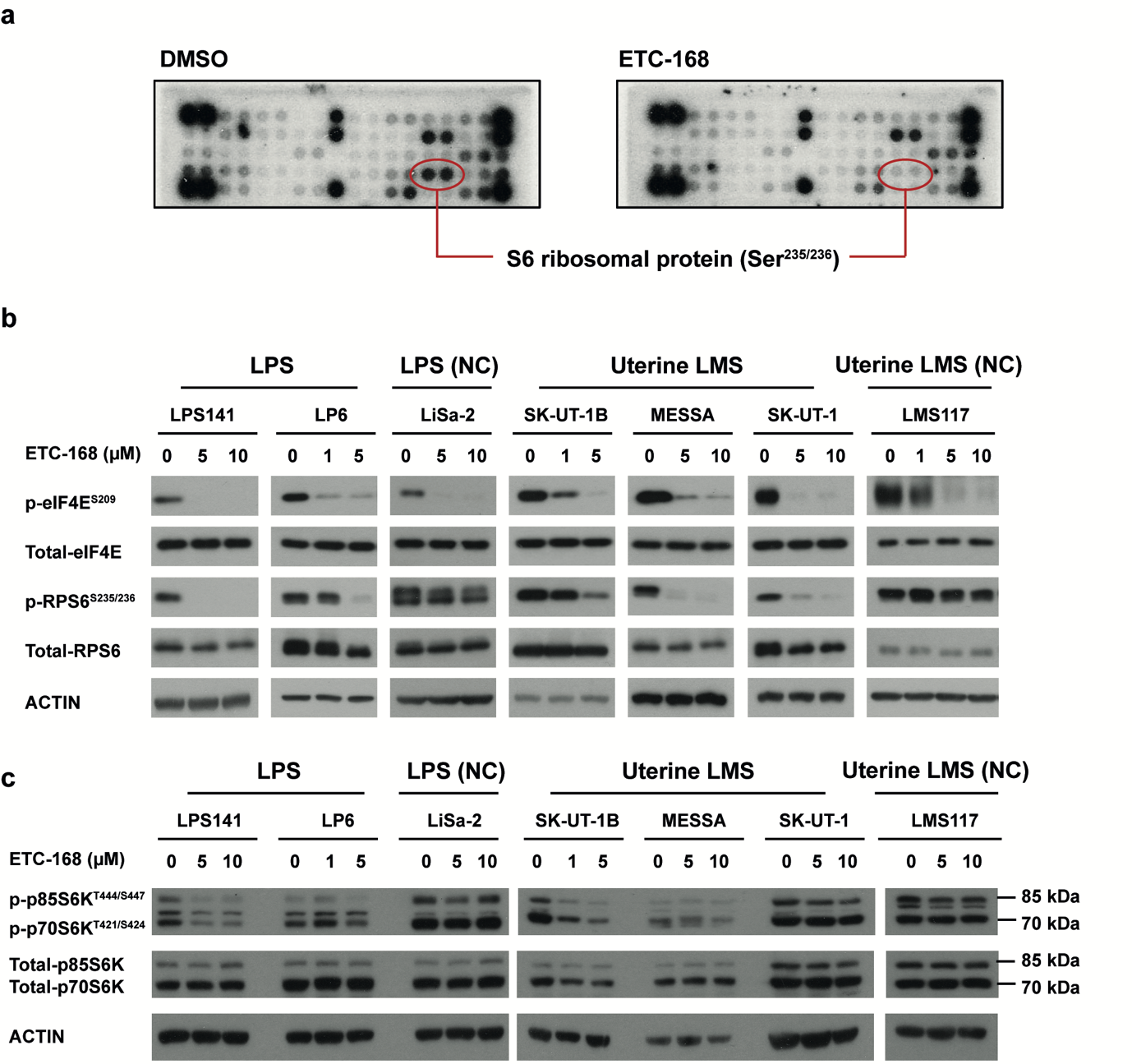


**Supplementary Figure S4.**

**Reduced phosphorylation of ribosomal protein S6 correlates with cellular responsiveness to ETC-168 treatment in de-differentiated LPS and uterine LMS cells.**

**a** Selective effect of ETC-168 treatment (10 μM) on p-RPS6 Ser^235/236^ in LPS141 cells. Whole cell lysates were harvested 8 hours after treatment and subjected to PathScan® RTK Signaling Antibody Array analysis. **b** Western blot analysis showing expression of eIF4E and RPS6 in response to ETC-168 treatment (24 hours) in LPS and uterine LMS cell lines. NC, negative control cells. **c** Western blot analysis of S6K expression in response to ETC-168 treatment (24 hours) in LPS and uterine LMS cell lines. NC, negative control cells.


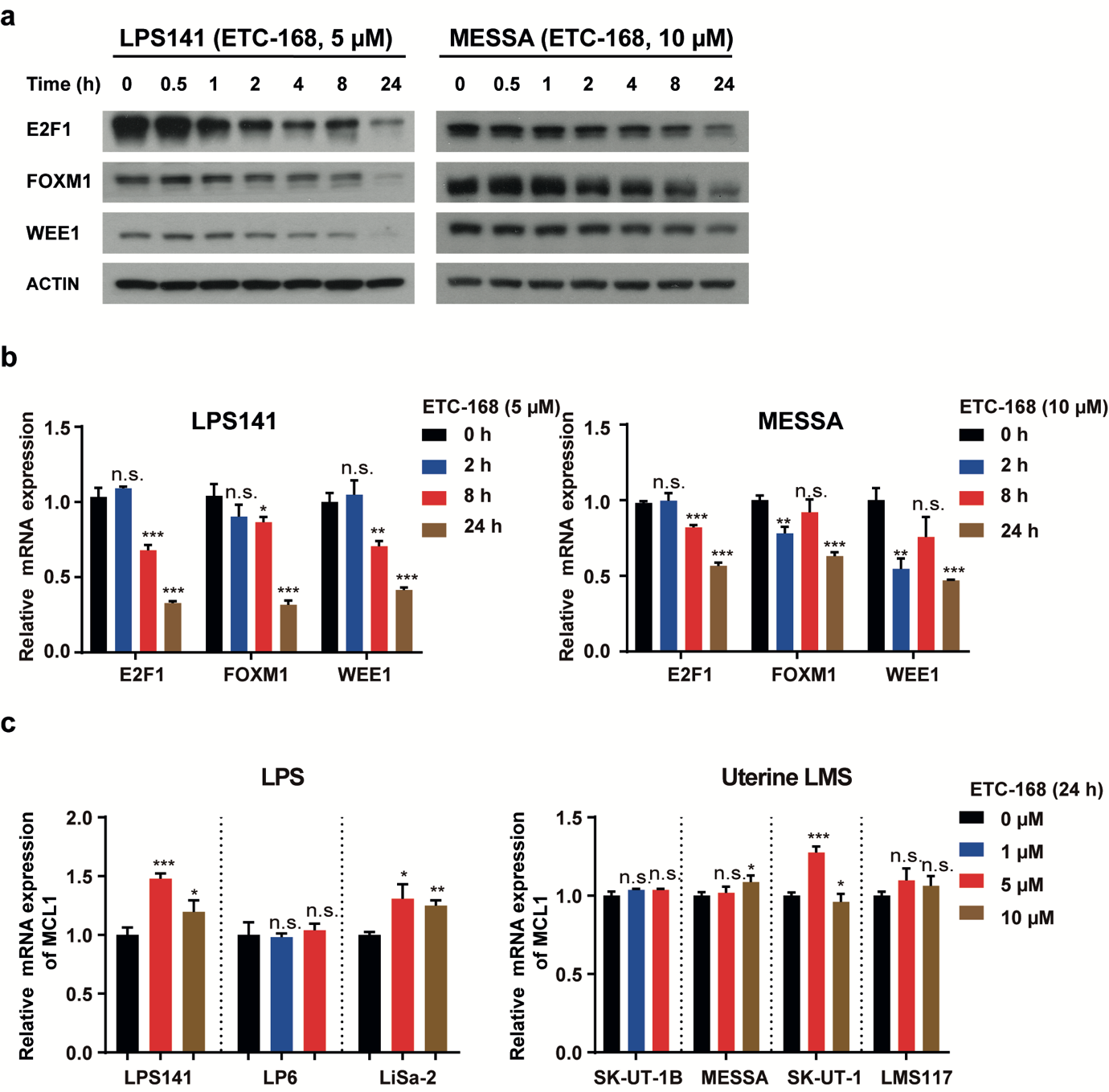


**Supplementary Figure S5.**

**ETC-168 regulates expression of E2F1, FOXM1, WEE1 and MCL1 in STS cells.**

**a and b** Time-course analysis of: **(a)** protein and **(b)** mRNA levels of E2F1, FOXM1 and WEE1 in LPS141 and MESSA cells in response to ETC-168 treatment. **c** Effect of ETC-168 treatment on MCL1 mRNA levels in LPS and Uterine LMS cells. Data of **b** and **c** are representative of three independent experiments and are shown as mean ± SD. Statistical significance is determined by one-way ANOVA. n.s., not significant; *: p<0.05; **: p<0.01; ***: p<0.001.

**
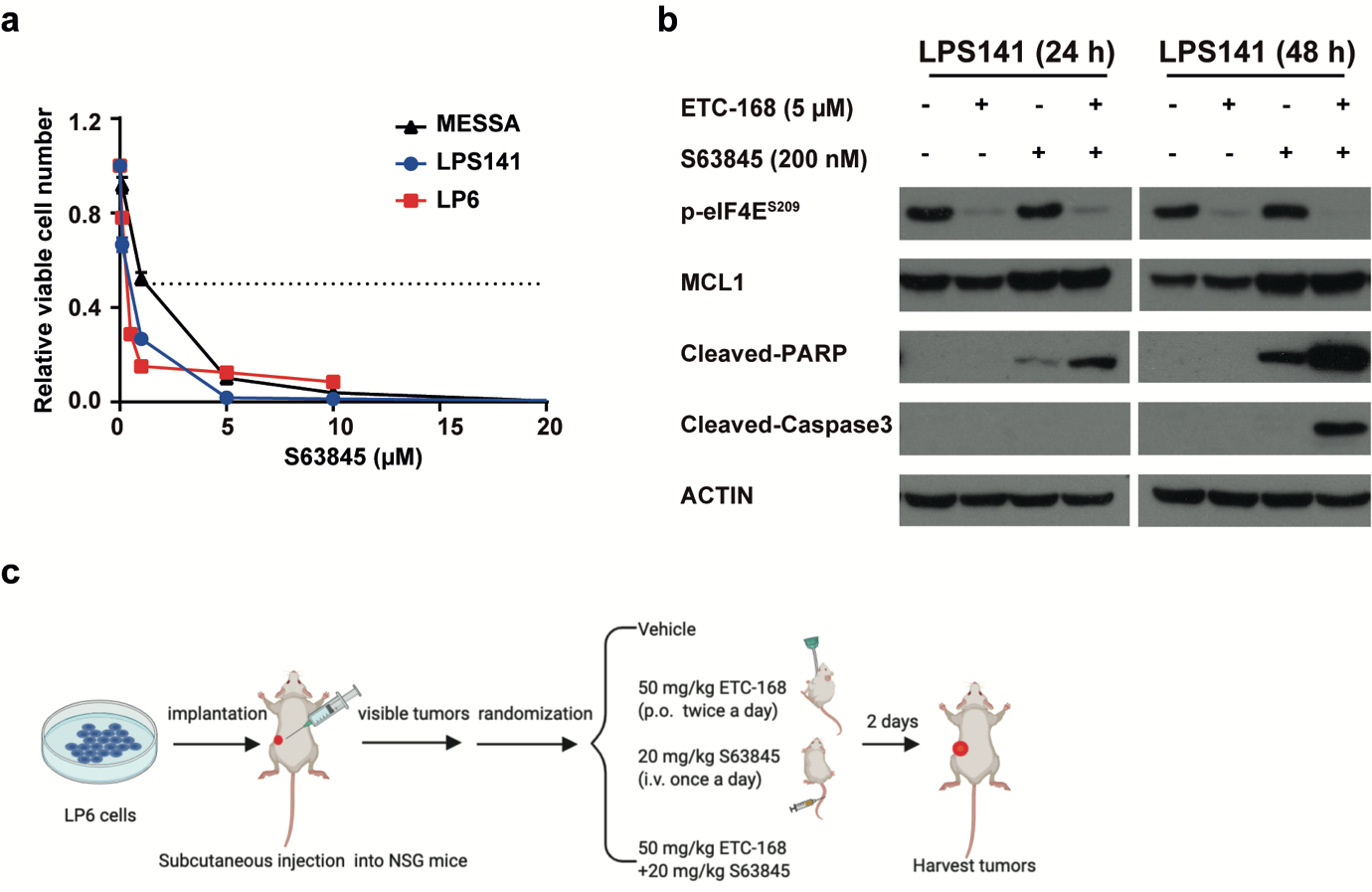
**

**Supplementary Figure S6.**

**Combination of ETC-168 and S63845 inhibits STS cell viability.**

**a** Dose-response curves showing efficacy of MCL1 inhibitor (S63845) against the viability of STS cells (72 hours). **b** Western blot showing the effect of ETC-168 and S63845 combination (24 hours and 48 hours) on expression of eIF4E and apoptosis-related proteins in LPS141 cells. **c** Schematic diagram showing the schedule of drug treatment for tumor-bearing NSG mice. Data of **a** are shown as mean ± SD (n=3).
